# Changes in Sales of E-Cigarettes, Cigarettes, and Nicotine Replacement Therapy Products Before, During, and After the EVALI Outbreak

**DOI:** 10.5888/pcd19.220087

**Published:** 2022-12-15

**Authors:** Xu Wang, Yoonsang Kim, Katrina F. Trivers, Michael A. Tynan, Sundar S. Shrestha, Sherry Emery, Mateusz Borowiecki, Karen Hacker

**Affiliations:** 1 Office on Smoking and Health, National Center for Chronic Disease Prevention and Health Promotion, Centers for Disease Control and Prevention, Atlanta, Georgia; 2 Public Health, National Opinion Research Center (NORC) at the University of Chicago, Chicago, Illinois; 3 National Center for Chronic Disease Prevention and Health Promotion, Centers for Disease Control and Prevention, Atlanta, Georgia

## Abstract

**Introduction:**

In 2019, an outbreak of e-cigarette, or vaping, product use–associated lung injury (EVALI) occurred in the US. We used Nielsen retail sales data to assess trends in sales of e-cigarettes, cigarettes, and nicotine replacement therapy (NRT) products before, during, and after the EVALI outbreak.

**Methods:**

Monthly unit sales of e-cigarettes, cigarettes, and NRT products overall and by product type were assessed during January 2019 through June 2020 by using an interrupted time series model. Two time points were specified at the period ending July 13, 2019, and the period ending February 22, 2020, to partition before, during, and after the outbreak period. Sales trends by aggregated state-level EVALI case prevalence (low, medium, and high) were assessed to investigate interstate variations in changes of sales coinciding with the EVALI outbreak.

**Results:**

Monthly e-cigarette sales increased 3.5% (*P* < .001) before the outbreak and decreased 3.1% (*P* < .001) during the outbreak, with no significant changes after the outbreak. Monthly cigarette sales increased 1.6% (*P* < .001) before the outbreak, decreased 1.8% (*P* < .001) during the outbreak, and increased 2.7% (*P* < .001) after the outbreak. NRT sales did not change significantly before or during the outbreak but decreased (2.8%, *P* = .01) after the outbreak. Sales trends by state-level EVALI case prevalence were similar to national-level sales trends.

**Conclusion:**

Cigarette and e-cigarette sales decreased during the EVALI outbreak, but no changes in overall NRT sales were observed until after the outbreak. Continued monitoring of tobacco sales data can provide insight into potential changes in use patterns and inform tobacco prevention and control efforts.

**Summary**

**What is known on this topic?**

Few studies have assessed trends in sales or use of tobacco products around the EVALI (e-cigarette, or vaping, product use–associated lung injury) outbreak, and none have assessed the changes in sales of nicotine replacement therapy (NRT) products around the outbreak.

**What is added by this report?**

This study identifies trends in sales of e-cigarettes, cigarettes, and NRT products before, during, and after the EVALI outbreak. US state sales trends were analyzed in relation to low, medium, and high EVALI case prevalence.

**What are the implications for public health practice?**

Continued monitoring of tobacco sales data can provide insight into potential changes in use patterns and inform tobacco prevention and control efforts.

## Introduction

Tobacco use remains the leading cause of preventable disease and death in the United States (1,2). Disease and death from tobacco use is overwhelmingly caused by combusted tobacco products (1). Since the first Surgeon General’s Report on smoking and health, cigarette smoking among US adults declined from about 43% in 1964 to 14% in 2019 (2,3). While the prevalence of cigarette smoking continues to decline among US adults and youth, the prevalence of e-cigarette use increased since their introduction to the US market in 2007, reaching a prevalence of 4.5% among adults and 20% among youth by 2019 (3,4). Although current use of e-cigarettes decreased in 2020 to 3.3% among US adults and 13% among youth (middle- and high-school students) (5,6), e-cigarettes have been the most commonly used tobacco product among US middle (grades 6–8) and high school (grades 9–12) students since 2014 (7).

E-cigarette aerosol can contain harmful and potentially harmful substances (8). Most e-cigarettes sold contain nicotine**, **a highly addictive drug that can harm brain development, which continues until about age 25 (9). E-cigarette use can also increase the risk of combustible cigarette use among youth and may cause sustained tobacco use among youth (10,11). Many people may consider e-cigarette use as a way to quit cigarette smoking (12,13). However, there is inadequate evidence to conclude that e-cigarettes, in general, increase smoking cessation, and e-cigarettes are not currently approved by the US Food and Drug Administration (FDA) as a smoking cessation aid (2). Cessation medications approved by the FDA for use in adults aged older than 18 include bupropion, varenicline, and 5 forms of nicotine replacement therapy (NRT; nicotine patch, gum, lozenge, inhaler, and nasal spray). Because e-cigarettes can harm the developing brain, e-cigarettes should never be used by youth, young adults, and women who are pregnant (8). Adults who do not use tobacco products should not start using e-cigarette, or vaping, products (2).

In 2019, an outbreak of e-cigarette, or vaping, product use–associated lung injury (EVALI) occurred in the US. Starting in August 2019, the Centers for Disease Control and Prevention (CDC), the FDA, and state and local health authorities investigated the nationwide EVALI outbreak. The number of EVALI cases reported to CDC peaked in September 2019 and then had a gradual and persistent decline (14). As of February 18, 2020, a total of 2,807 hospitalized EVALI cases were reported to CDC from all 50 states, the District of Columbia, the US Virgin Islands, and Puerto Rico. Investigative results indicate that tetrahydrocannabinol (THC)-containing e-cigarette, or vaping, products — particularly those obtained from informal sources like family, friends, or in-person or online dealers — were linked to most EVALI cases, and vitamin E acetate, an additive in THC devices, played a major role in the outbreak (14,15). While most EVALI patients (82%) reported using THC-containing products and more than half (57%) reported also using nicotine-containing products, about 1 in 7 patients (14%) reported exclusive use of nicotine-containing products (14).

Because of the initial uncertainty around the cause of EVALI (16), the outbreak may have prompted changes in tobacco use and cessation behaviors. Although not necessarily reflective of use patterns, retail sales data may provide a useful barometer to provide insight into changes in e-cigarette use, cigarette smoking, and tobacco use cessation patterns. Some studies have assessed trends in sales or use of cigarettes and e-cigarettes (15,17–19). However, these studies did not cover the period of the EVALI outbreak. One study did assess the e-cigarette unit sales in the US during September 2014 through May 2020, which covered the EVALI period (20), but like other studies (15,17–19), it did not assess the trends in sales or use of NRT around the EVALI outbreak. Our study used 4-week aggregate retail sales data to identify emerging trends in sales of e-cigarettes, cigarettes, and NRT products before, during, and after the outbreak. Sales trends by states with low, medium, and high EVALI case prevalence were also assessed to investigate variations in changes of sales coincident with the EVALI outbreak at the state level.

## Methods

### Data

Scanner data of US national and state tobacco retail stores were obtained from The Nielsen Company (Nielsen). Sales data represent sales in the 48 contiguous US states and the District of Columbia and do not include Hawaii and Alaska. Nielsen provided data on unit sales and product characteristics for each Universal Product Code (UPC) at the national and state levels in 4-week aggregates from the 4-week period ending January 26, 2019, through the period ending June 13, 2020. Nielsen data represent sales in convenience stores (chain, franchise, and independent, with and without provisions for gasoline) and sales in Nielsen’s “All Outlet Combined” (AOC) channel, which represents sales in food/grocery stores, pharmacies, mass merchandisers (eg, Walmart), club stores (eg, Sam’s Club), discount/dollar stores, and US military commissaries. Internet and vape shop sales were not included.

### Measures

E-cigarette and NRT products were categorized on the basis of UPC-specific information on the product description, form, and target use. When the UPC-specific information provided by Nielsen was insufficient, products were identified and categorized manually via extensive online searches (ie, using brand and vendor websites).

#### E-cigarette and cigarette unit sales

E-cigarettes were categorized into 3 types: disposable e-cigarettes, pods/cartridges (including rechargeable devices with pods/cartridges), and e-liquid bottles for refillable devices. Items with rechargeable devices only, accessories, and uncategorized products were excluded from the analysis. E-cigarette unit sales were standardized for aggregation: 1 unit of e-cigarette products equals 1 disposable e-cigarette, 1 e-liquid bottle, or 5 prefilled pods/cartridges (19). Cigarette units were standardized as 1 unit equals 1 pack of cigarettes, which typically contains 20 to 25 cigarettes.

#### Over-the-counter–NRT unit sales

Nielsen data include only sales of over-the-counter (OTC) NRTs. Our study focused on the 3 FDA-approved OTC-NRTs: nicotine gum with 2 mg or 4 mg strength, nicotine lozenge with 2 mg or 4 mg strength, and nicotine patch with 7 mg, 14 mg, or 21 mg strength (21). NRT products with unusual nicotine strength (<0.01% of total OTC-NRT dollar sales) were excluded. OTC-NRT products were standardized as 1 unit equals 1 nicotine patch with 7 mg strength, 1 nicotine lozenge with 2 mg strength, or 1 nicotine gum with 2 mg strength.

### Analysis

An interrupted time series (ITS) model (22) was used to assess the significance and magnitude of changes in unit sales before (January–June 2019), during (July 2019–February 2020), and after (March–June 2020) the EVALI outbreak (23). Two time points were specified at the period ending July 13, 2019, and the period ending February 22, 2020, to partition the period. The linear regression of unit sales in log-scale with linear splines was used for the ITS model. Total unit sales were calculated by 4-week period (hereinafter referred to as “month”). Model-based monthly change in unit sales for before, during, and after the outbreak were estimated, and the associated *P* values were reported for testing whether these relative changes per month were significant (*P* < .05). We generated graphs visualizing observed and model-based unit sales around the outbreak, with indication of other events that were potentially associated with tobacco sales. A sensitivity analysis was conducted to control for the confounding factor of FDA’s flavored e-cigarette enforcement guidance that took effect on February 6, 2020. The guidance was to remove pod/cartridge-based, non–menthol-flavored e-cigarettes from the market, which could have a direct impact on e-cigarette sales and other tobacco product sales.

To assess the variation in unit sales across states, states were grouped on the basis of the distribution of state-level EVALI incidence per million population (24) (Appendix Table 1): low (≤5.0), medium (5.1–9.9), and high (≥10.0). Total unit sales for each state were adjusted for state population size. Total unit sales per 100,000 state population were calculated and summed within each of the groups. The relative amount of monthly change for each state group was estimated using the ITS model.

## Results

The highest monthly unit sale of e-cigarettes was 17,660,488 in August 2019, which occurred in the initial months of the “during” EVALI period, while the lowest was 13,086,427 in February 2020, the last month of the “during” EVALI period (Figure). The average monthly unit sales of e-cigarettes were 15,810,024 before the outbreak; 15,522,976 during the outbreak; and 13,953,473 after the outbreak. The decline in average unit sales represents a −1.8% relative percentage change from before the outbreak to during and a −10.0% relative percentage change from during the outbreak to post-outbreak. Overall, e-cigarette unit sales increased 3.5% per month (*P* < .001) before the outbreak and declined 3.1% per month (*P* < .001) during the outbreak, with no significant changes (0.8% per month, *P* = .44) after the outbreak (Table 1). By product type, pod/cartridge was the dominant product purchased throughout the time frame. Before the outbreak, pod/cartridge sales increased 4.0% per month (*P* < .001). During the outbreak, disposable product sales increased 4.0% per month (*P* < .001), e-liquids sales decreased 30.7% (*P* < .001), and pod/cartridge sales decreased 4.1% (*P* < .001).

**Figure:**
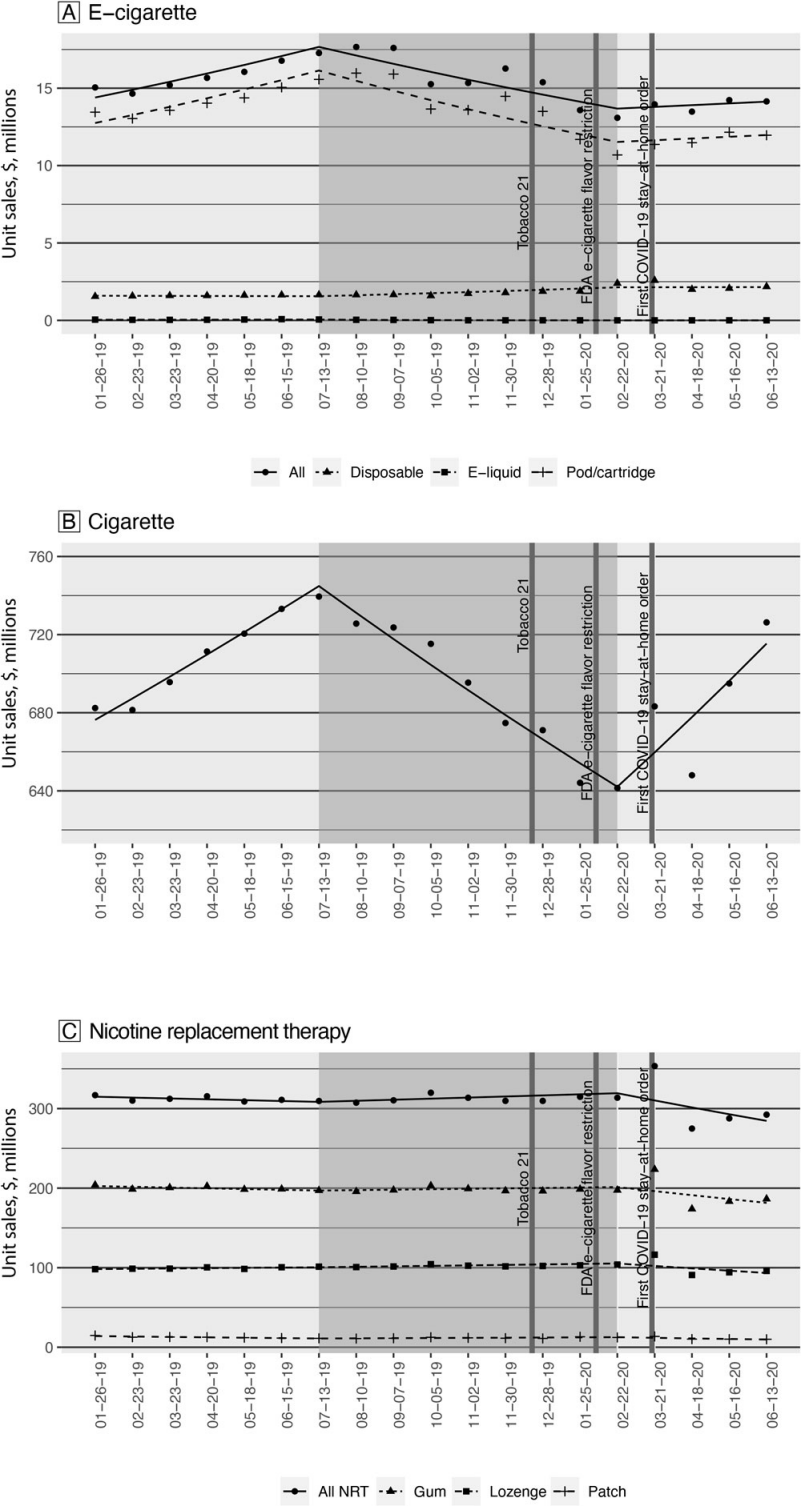
Monthly unit sales of A) e-cigarettes, B) cigarettes, and C) over-the-counter (OTC)–NRT, by product type before, during, and after the EVALI outbreak. Geometric shapes indicate observed sales, and lines indicate predicted sales based on the interrupted time series (ITS) model. The dates on the x axis are ending dates of each 4-week period. The dark-gray box indicates the period “during” the outbreak. Unit sales were calculated per 4-week period. One unit of e-cigarette products equals 1 disposable e-cigarette, 1 e-liquid bottle, or 5 prefilled pods/cartridges. Cigarette units were standardized as 1 unit equals 1 pack of cigarettes, which typically contains 20 to 25 cigarettes. OTC-NRTs included nicotine patch, gum, and lozenge. One unit of NRT products equals 1 nicotine patch with 7 mg nicotine strength, or 1 nicotine lozenge or 1 nicotine gum with 2 mg nicotine strength each. Abbreviations: FDA, Food and Drug Administration; NRT, nicotine replacement therapy; Tobacco 21, passage of federal legislation that increased the legal tobacco purchase age to 21.

**Table 1:** Model-Based Average Monthly Changes^a^ in Unit Sales of E-Cigarettes, Cigarettes, and OTC-NRT^b^ Products Before, During, and After the EVALI Outbreak, US, 2019^c^

Product type	Before EVALI		During EVALI		After EVALI	
	Monthly change, %	*P* value	Monthly change, %	*P* value	Monthly change, %	*P* value
**E-cigarettes**	3.5	<.001	−3.1	<.001	0.8	.44
Disposable	−0.3	.79	4.0	<.001	0.2	.94
Pod/cartridge	4.0	<.001	−4.1	<.001	1.0	.47
E-liquid	5.6	.24	−30.7	<.001	12.1	.12
**Cigarettes**	1.6	<.001	−1.8	<.001	2.7	<.001
**NRT**	−0.3	.62	0.4	.35	−2.8	.01
Gum	−0.5	.50	0.3	.55	−2.6	.02
Lozenge	0.4	.58	0.6	.20	−3.0	.006
Patch	−4.0	<.001	1.7	.01	−6.3	<.001

Abbreviations: EVALI, e-cigarette or vaping product use–associated lung injury; NRT, nicotine replacement therapy; OTC, over-the-counter.

a Average monthly changes were derived by using interrupted time series (ITS) model estimates, and *P* values were calculated based on *z* test statistic to test for whether they differ from zero (ie, no change).

b OTC-NRT included nicotine patch, gum, and lozenge.

c Significance was determined by a 2-sided test with *P* < .05.

The highest monthly unit sale of cigarettes was 739,473,399 in July 2019, which was the first month of the “during” EVALI period; the lowest was 641,445,399 in February 2020, which was the last month of the “during” EVALI period (Figure). The average monthly unit sales of cigarettes were 709,145,992 before the outbreak; 686,417,973 during the outbreak; and 688,120,050 after the outbreak. The change in average unit sales represents a 3.2% decrease from before the outbreak to during the outbreak and a 0.2% increase from during the outbreak to post outbreak. The monthly unit sales of cigarettes increased 1.6% (*P* < .001) per month before the outbreak, decreased 1.8% (*P* < .001) per month during the outbreak, and increased 2.7% (*P* < .001) per month after the outbreak (Table 1).

The highest monthly unit sale of OTC-NRT products was 353,465,526 in March 2020 which was the first month of “after” the EVALI period. The average monthly unit sales of NRT products were 312,026,992 before the outbreak; 312,418,838 during the outbreak; and302,114,005 after the outbreak (Figure). The change in average unit sales represents a 0.1% decrease from before the outbreak to during and a 3.3% decrease from during the outbreak to post outbreak. Overall, NRT sales were generally steady with no changes before or during the outbreak, followed by a 2.8% decrease per month (*P* = .01) after the outbreak. By product type, nicotine gum was the most commonly purchased OTC-NRT product throughout the study period. Monthly unit sales of nicotine gum and lozenge showed no significant changes before or during the outbreak, while monthly unit sales for nicotine patches decreased 4.0% (*P* < .001) before the outbreak and increased 1.7% (*P* = .01) during the outbreak. After the outbreak, all 3 types of OTC-NRT products decreased in monthly unit sales: 2.6% decrease for nicotine gum (*P* = .02), 3.0% decrease for nicotine lozenge (*P* = .006), and 6.3% decrease for nicotine patch (*P* < .001).

Estimates from the sensitivity analysis showed results largely consistent with those from the main analysis, except for pod/cartridge and disposable e-cigarettes (Appendix Table 2). After controlling for the effect of FDA’s flavored e-cigarette enforcement guidance, pod/cartridge sales increased 3.0% per month (*P* = .04) and disposable product sales decreased 4.0% per month (*P* = .01) after the outbreak.

Across all products, the medium-EVALI prevalence states had higher per-capita unit sales than low- or high-EVALI prevalence states throughout the study period. The assessment of unit sales by state-level EVALI prevalence showed trends similar to national-level sales trends. In general, the sales trends over time and model-based monthly change for unit sales per 100,000 people were similar across low-, medium-, and high-EVALI prevalence states (Table 2).

**Table 2:** Model-Based Average Monthly Changes^a^ in Unit Sales of E-Cigarettes, Cigarettes, and OTC-NRT^b^ Products by State-Level EVALI Case Counts Per Million Population^c^ Before, During, and After the EVALI Outbreak, US, 2019^d^

State-level EVALI case counts	Before EVALI		During EVALI		After EVALI	
	Monthly change, %	*P* value	Monthly change, %	*P* value	Monthly change, %	*P* value
**E-cigarette unit sales per 100,000 population**						
High	3.6	<.001	−4.2	<.001	1.3	.15
Medium	5.1	<.001	−3.0	<.001	0.4	.84
Low	3.6	.002	−3.2	<.001	0.7	.63
**Cigarette unit sales per 100,000 population**						
High	2.6	<.001	−2.4	<.001	3.9	<.001
Medium	2.1	<.001	−2.0	<.001	3.0	<.001
Low	1.7	<.001	−2.2	<.001	3.2	<.001
**NRT unit sales per 100,000 population**						
High	0.4	.55	0.1	.79	−2.5	.03
Medium	0.1	.91	0.2	.73	−2.1	.06
Low	−0.1	.84	0.3	.58	−3.0	.008

Abbreviations: EVALI, e-cigarette or vaping product use–associated lung injury; NRT, nicotine replacement therapy; OTC, over-the-counter.

a Average monthly changes were estimated using interrupted time series (ITS) model.

b OTC-NRT included nicotine patch, gum, and lozenge.

c States were grouped based on state-level EVALI case counts per million population: low (<5), medium (5–9), and high (≥10).

d Significance was determined by a 2-sided test with *P* < .05.

## Discussion

The results from this study suggest that e-cigarette, cigarette, and NRT product sales changed coincident with the EVALI outbreak. Overall, e-cigarette unit sales increased before the outbreak, declined during the outbreak, and did not significantly change after the outbreak as of June 2020. The average monthly unit sales of e-cigarettes were lowest after the outbreak compared with the before and during outbreak periods. These findings suggest that e-cigarette sales declined significantly during the outbreak and remained at a lower volume after the outbreak through mid-June 2020 as compared with the pre-outbreak sales volume. Our results are well aligned with other studies of sales data, which showed that e-cigarette sales started to decline from summer 2019 through the early months of 2020 (18,20). Estimates from survey data also showed that from 2019 to 2020, e-cigarette use prevalence decreased from 27.5% to 19.6% among high-school students and decreased from 10.5% to 4.7% among middle-school students (6,25). E-cigarette use prevalence for adults aged 18 years or older also decreased from 3.9% in quarter one 2019 to 3.6% in quarter two 2020 (26). One potential explanation for observed declines in e-cigarette sales is that the EVALI outbreak raised the public’s awareness about e-cigarette safety, which led to significantly reduced e-cigarette purchases during the outbreak and which continued after the outbreak. Other factors that may have contributed to the observed changes in e-cigarette sales include the passage of federal legislation that increased the legal tobacco purchase age to 21 (ie, Tobacco 21 policy) on December 20, 2019, and FDA’s flavored e-cigarette enforcement guidance. Results from the sensitivity analysis did indicate that after the outbreak, FDA’s flavored e-cigarette enforcement guidance was associated with decreased sales of pod/cartridge products but increased sales of disposable products, although the overall association with e-cigarette sales was not significant. In addition, flavored tobacco product restrictions of varying types at state and local levels may have further discouraged the use of some types of e-cigarettes, especially among youth since they are particularly attracted to flavored e-cigarette products (8). Despite the federal, state, and local regulation of flavored tobacco products, some flavored products continued to be available across the US, including flavored cigars, menthol-flavored cigarettes, and cartridge e-cigarettes and disposable e-cigarette products in both menthol and other flavors (20). Youth use of tobacco products, including e-cigarettes, is unsafe. Adults who want to quit smoking should seek proven methods to quit, such as behavioral counseling and FDA-approved cessation medications (2).

The cigarette sales trend indicates that cigarette unit sales increased before the outbreak, decreased during the outbreak, and recovered after the outbreak with an even faster rate of increase compared with the pre-outbreak period. The observed sales trend in cigarettes may be partially explained by the substitution relationship between cigarettes and e-cigarettes (27,28). Some e-cigarette users may have switched to cigarette use as they reduced their e-cigarette consumption precipitated by the EVALI outbreak. Additionally, the concurrence of the emergence of the COVID-19 pandemic in the post-EVALI period may have also contributed to the rise of cigarette sales starting in March 2020. During the initial months of the COVID-19 pandemic, people may have engaged in stockpiling behaviors in anticipation of restrictions. As states and communities issued stay-at-home orders and essential businesses closed, people spent more time at home, which possibly gave them more opportunities to use tobacco products when not in public places (29). Cigarette smoking may be falsely perceived by some as a stress reducer, which could possibly have increased cigarette demand during the COVID-19 pandemic. More research is needed to fully explore how the COVID-19 pandemic affected tobacco purchasing and use behaviors, including tobacco use initiation and relapse, tobacco product switching, increased or decreased use intensity, and cessation.

Overall, OTC-NRT unit sales were generally steady with no changes before or during the outbreak but decreased after the outbreak. However, nicotine patch unit sales decreased before the outbreak and increased during the outbreak. This may indicate increased cessation treatment–seeking behaviors during the EVALI outbreak. The subsequent decrease of OTC-NRT sales in early 2020 may indicate a decrease in cessation treatment–seeking behaviors, which could be due to several factors including potential decreases in cessation behaviors during the emergence of the COVID-19 pandemic (30).

The assessment of unit sales by state-level EVALI prevalence showed that the medium-EVALI prevalence states had higher per-capita unit sales than low- or high-EVALI prevalence states in general. This may be due to the few large states included in this group, such as Florida, Michigan, New York, North Carolina, Ohio, Pennsylvania, and Texas. These states have the highest sales levels among all states. Overall, sale trends by state-level EVALI prevalence showed similar trends to national-level sales trends across low-, medium-, and high-EVALI prevalence states.

### Limitations

This study has limitations. First, internet and vape shop sales were not included in the data. Therefore, unit sales reported in our study could underestimate the total sales volume. Second, we did not account for a few federal-, state-, or local-level tobacco control policy changes implemented during or adjacent to the study period (eg, state and local regulations of flavored tobacco products, state and local cigarette and e-cigarette tax increases, and the passage and subsequent enforcement of the federal Tobacco 21 policy). In addition, the after-EVALI period included the beginning of the COVID-19 pandemic. These policies and events may have affected the estimated rate of change in product sales during periods around the EVALI outbreak. However, results from sensitivity analysis controlling for FDA’s flavored e-cigarette enforcement guidance show largely consistent results from the main analysis. Because of the limited number of data points (19 four-week periods) during our study period, controlling additional confounding factors could reduce the precision of the model estimates. Third, this study did not include data on unit sales of prescription cessation medications. Therefore, unit sales trends related to smoking cessation medications are not fully captured. Fourth, national-level unit sales reported in this study only represented sales in the 48 contiguous US states and District of Columbia because Nielsen sales data did not include data from Hawaii and Alaska. Finally, sales data cannot be extrapolated to be estimates of tobacco use and cessation. Despite these limitations, the data presented here reflect the most up-to-date and complete picture of sales trends of e-cigarette, cigarette, and NRT products before, during, and after the EVALI outbreak.

### Conclusion

Our findings suggest that changes in tobacco and NRT product sales occurred relative to the timing of the EVALI outbreak. E-cigarette sales increased before, decreased during, and stabilized after the outbreak at sales volumes lower than those before the outbreak. In contrast, cigarette sales increased before, decreased during, and increased after the outbreak while nicotine patch sales increased during the outbreak, but overall NRT sales decreased after the outbreak. These results suggest that additional public health efforts, including promoting the use of tobacco quitlines and increasing awareness of the benefits of FDA-approved cessation medications, may be needed. Public health outbreaks related to tobacco products can affect product use and quitting. Monitoring tobacco and NRT product sales trends can give insight into potential changes in use patterns in relation to public health events, such as the EVALI outbreak. Such information can help guide and inform public health action and future outbreak response efforts at the national, state, and local levels.

## Appendix Supplemental Tables

**Supplemental Table 1:** Rate Per 1 Million Population of Hospitalized Cases and Deaths of EVALI, by US State^a^, August 2019**–**February 2020

State	Cases per 1 million population	EVALI group^b^
Utah	40.8	High
North Dakota	26.3	High
Minnesota	24.4	High
Delaware	20.7	High
Indiana	19.1	High
Illinois	16.9	High
Iowa	16.5	High
Wisconsin	16.2	High
Massachusetts	16.1	High
South Dakota	14.7	High
Connecticut	14.3	High
New Jersey	12.1	High
Virginia	11.3	High
New Mexico	11.0	High
Tennessee	10.8	High
Pennsylvania	9.4	Medium
Texas	8.5	Medium
Maryland	8.3	Medium
Ohio	8.0	Medium
Kansas	7.9	Medium
Nebraska	7.8	Medium
West Virginia	7.8	Medium
New York	7.7	Medium
Arkansas	7.6	Medium
Louisiana	7.3	Medium
North Carolina	7.2	Medium
South Carolina	7.1	Medium
Idaho	6.8	Medium
Michigan	6.7	Medium
Rhode Island	6.6	Medium
Montana	6.6	Medium
Missouri	6.5	Medium
Vermont	6.4	Medium
Florida	5.6	Medium
Oregon	5.5	Medium
Wyoming	5.2	Medium
New Hampshire	5.2	Medium
California	5.0	Low
Kentucky	4.9	Low
District of Columbia	4.3	Low
Georgia	4.0	Low
Maine	3.7	Low
Washington	3.6	Low
Mississippi	3.3	Low
Alabama	3.1	Low
Arizona	2.9	Low
Nevada	2.3	Low
Oklahoma	1.5	Low
Colorado	1.4	Low

Abbreviation: EVALI, e-cigarette or vaping product use–associated lung injury.

^a^ Data were shown for the 48 contiguous US states and the District of Columbia and do not include Hawaii and Alaska.

^b^ To assess the variation in unit sales across states, states were grouped on the basis of the distribution of state-level EVALI incidence per million population: low (≤5.0), medium (5.1–9.9), and high (≥10.0).

**Supplemental Table 2:** Sensitivity Analysis^a^ Results of Model-Based Average Monthly Changes^b^ in Unit Sales of E-Cigarettes, Cigarettes, and OTC-NRT^c^ Products Before, During, and After the EVALI Outbreak, US, 2019^d^

Product type	Before EVALI		During EVALI		After EVALI	
	Monthly change, %	*P* value	Monthly change, %	*P* value	Monthly change, %	*P* value
**E-cigarettes**	3.3	<.001	−2.7	<.001	2.8	.17
Disposable	0.5	.55	2.0	.004	−4.0	.01
Pod/cartridge	3.6	<.001	−3.3	<.001	3.0	.04
E-liquid	7.0	.15	−32.7	<.001	4.9	.59
**Cigarettes**	1.6	<.001	−1.9	<.001	2.7	<.001
**NRT**	−0.2	.75	0.2	.78	−3.4	.01
Gum	−0.4	.61	0.0	.95	−3.1	.03
Lozenge	0.5	.47	0.3	.65	−3.6	.007
Patch	−3.8	.002	1.4	.09	−6.8	<.001

Abbreviations: EVALI, e-cigarette or vaping product use–associated lung injury; NRT, nicotine replacement therapy; OTC, over-the-counter.

^a^ Controlled for the potential confounding factor of the Food and Drug Administration’s flavored e-cigarette enforcement guidance. A binary indicator equal to 1 for February 23, 2020, through June 13, 2020, was used to indicate the enforcement guidance was in effect and 0 before February 23, 2020.

^b^ Average monthly changes were estimated using interrupted time series (ITS) model.

^c^ OTC-NRT included nicotine patch, gum, and lozenge.

^d^ Significance was determined by a 2-sided test with *P* < .05.
